# Public engagement professionals: Exploring ethical tensions in communication, engagement and co-creation

**DOI:** 10.1177/09636625261440854

**Published:** 2026-05-05

**Authors:** Clare Wilkinson, Aleksandra Stelmach, Michael Parker, Milly Farrell

**Affiliations:** 1 UWE Bristol, UK; 2 University of Exeter, UK; 3 University of Oxford, UK

**Keywords:** co-creation, ethics, professionalisation, public engagement, science communication

## Abstract

Recent decades have seen the rising establishment of public engagement professionals supporting communication and engagement in the contexts of science and health research. This article explores how public engagement professionals consider the ethical dimensions of their work. Based on analysis of 17 interviews with science communication and public engagement specialists at UK academic institutions and in practice settings, we examine the ways in which they frame ethical issues associated with engagement, as well as how they frame both publics and researchers. In doing so, we explore two tensions that together highlight how the rising popularity of engagement methods, including engaged research, can increase awareness of the need to ethically scrutinise engagement and associated practices. We also argue that public engagement professionals can further contribute to and advance academic discussion around engagement, arguing that ongoing research *with* practitioners is beneficial for academic understanding of communication, engagement and engaged research.

## 1. Introduction

In recent decades, there has been significant investment in public communication, engagement and co-creation of research in the United Kingdom, as well as internationally and particularly around STEM (Science, Technology, Engineering, Mathematics) subjects (Chilvers, 2017; Davies and Avkıran, 2025; Pellegrini et al., 2025; Watermeyer, 2015; Watermeyer and Rowe, 2021). This has led to the development of dedicated funding initiatives, training and, increasingly, the embedding of engagement practices within research itself through Responsible Research and Innovation (RRI) and similar initiatives (e.g. European Commission, 2025; UKRI, 2023). Yet conversations around ethical aspects of engagement have lagged behind such investments (Wilkinson et al., 2025), though ethical issues associated with science often feature in the communication of research (Medvecky, 2018; Priest et al., 2018).

In this article, we adopt the UK National Coordinating Centre for Public Engagement’s definition of engagement. This states,

Public engagement describes the myriad of ways in which the activity and benefits of higher education and research can be shared with the public. Engagement is by definition a two-way process, involving interaction and listening, with the goal of generating mutual benefit. (NCCPE, 2026)

Within this description of engagement fall three broad types of communication: ‘inspiring and informing’, ‘consulting’ and ‘collaborating’. ‘Inspiring and informing’ aligns with definitions of science communication, also a sometimes-contentious concept to define, but broadly seen to relate to informing, raising awareness of and involving people beyond the scientific community, in science-related topics to tackle societal challenges (Science Europe, 2026). While the latter engagement type, ‘collaborating’, also relates to the concept of ‘co-creation’ and/or ‘co-production’, terms that can have varied definitions but which we use here to describe the involvement of citizens or communities in the research process itself, for example in contributing to the design of research questions, shaping data collection tools and/or producing communication outputs (Wilkinson and Weitkamp, 2026).

Research that has been conducted on both the engagement landscape and its ethical dimensions (e.g. Medvecky and Leach, 2019; Priest et al., 2018), has often been constructed around the dyad of researchers/communicators versus publics and their many interactions. Scholarly contributions on engagement, especially those more theoretically inclined, focus mainly on scientists or researchers as people driving and conducting engagement. By focusing on researchers’ actions, goals or intentions and the impact of that on publics and their reactions to engagement activities, the literature often concentrates predominantly on two sets of players, so to speak, in the engagement game and the power struggles and dilemmas of engagement from the perspective of publics and/or scientists, particularly the need for a two-way rather than deficit approach to engagement (see Simis et al., 2016 for further discussion of the deficit model).

Here, we focus on an additional set of actors who facilitate and/or conduct a large bulk of engagement activities and whose voice is not as often heard in scholarly articles – that of communication/engagement experts, or as we describe them, public engagement professionals (PEPs). We examine, via semi-structured interviews, the ways they frame engagement, specifically its ethical issues, as well as the ways that they frame both publics *and* researchers as participants in engagement/communication processes, providing a set of novel and nuanced perspectives that also highlight emerging tensions in conducting ‘ethical’ engagement and recognising professionalisation among engagement practitioners.

## 2. Literature review

There is currently limited awareness of the context of ethics within science communication and public engagement (Medvecky and Leach, 2017; Wilkinson et al., 2025). The limited resources available refrain from providing specific ethical standards or guidance (Medvecky and Leach, 2019; Priest et al., 2018; Wray, 2021). Furthermore, given that much public engagement activity takes place in practitioner settings (such as museums, science centres and other informal learning spaces), or via both traditional and social media, a large swath of engagement work likely takes place with limited ethical stop checks or reflection. Though some would argue a normative ethical framework for research engagement is undesirable (Medvecky and Leach, 2017), this remains underexplored. Emerging work has started to consider the context of ethics associated to topics including citizen science, where people are directly involved in the gathering or analysis of research (Groot and Abma, 2022), relationships between research communication and public relations (Roberson, 2020) and researchers’ communication training and/or competencies (Baram-Tsabari and Lewenstein, 2022; Seethaler et al., 2019), to name just a few areas, but there remain considerable gaps.

Changes to the communication landscape in recent decades have altered the ecosystem of those communicating science and research (Wilkinson et al., 2022). Researchers and science journalists are now just two communities in a landscape that includes research centres, universities, funding bodies, scientific publishers, science centres and museums, charities and amateur producers creating content and generating engagement (Lee, 2014; Letourneau, 2018; Weitkamp et al., 2021). With enhanced investment in engagement in some countries, including the United Kingdom, we have seen the rising establishment of PEPs, among this broader ecosystem. The National Coordinating Centre for Public Engagement (NCCPE, 2025) defines PEPs as those ‘with roles relating to public engagement’, including people working for universities, other organisations and as freelancers. PEPs can be working in central teams, or distributed to departments, and include professionals across all forms of engagement, including patient engagement, public engagement, outreach, impact, community engagement, civic engagement, evaluation and more. PEPs may also be independently or jointly applying for public engagement funding with researchers.

Traditionally, the term ‘profession’ is associated with vocations with a shared theoretical approach, networks, training, a code of ethics and a commitment to serve publics, elements of which we can increasingly see associated with PEPs (Barry and Legacy, 2022; Brunner, 2017). In the United Kingdom, the rising prominence of this role means PEPs are now supported by dedicated networks, such as the Association of Consultation and Engagement Professionals, and have some professional structures, like attribute frameworks. However, despite the rising professionalisation of PEPs, they have received limited focus in academic research. Watermeyer and Rowe (2021) posit that there may be multiple factors at play in this research gap, including long-standing hierarchies between academics and other professional service colleagues (which PEPs are frequently identified as), which can leave PEPs’ contributions under-recognised and even ‘invisible’ (Bherer et al., 2017a) in some academic and engagement contexts.

Although academic research focused on PEPs is limited, it is not non-existent. Almost two decades ago, Bauer et al. (2007) described the emergence of a new sector of ‘angels’ mediating between publics, science, industry and policymaking, though their observations were restricted to the need to scrutinise such developments. Chilvers (2007, 2017) work also highlighted the neglect of academic attention on the ‘actors’ shaping public engagement in science, emphasising the importance of practitioners in constructing participatory processes and reflecting on its challenges. A number of articles have since focused on the experiences of PEPs in developing engagement activities, case studies and/or frameworks (e.g. Altman et al., 2023; Christensen, 2019; Holmes et al., 2019; Koivumäki and Wilkinson, 2020; Lee, 2014; Pinto et al., 2018; Salmon and Roop, 2019; Wilkinson et al., 2010) often examining the practical issues they encounter but also occasionally making observations about attitudes and experiences in PEPs’ roles. Some of these accounts also connect these issues to long-standing concerns related to gaps between research and practice (Salmon and Roop, 2019) or briefly touch on the role of ethics (Christensen, 2019).

Besley et al. (2021) called for greater attention to the role of communication professionals in fostering high-quality public engagement, finding in their study that there was a need to better understand the relationships between researchers and professional communicators. Although their work suggested the need for an enhanced understanding of organisational roles in engagement, particularly as PEPs were seen to hold a degree of influence over researchers’ perspectives on engagement and its ethical incentives, the data primarily came from the perspectives of scientists (Besley et al., 2021). Thus, it was based on perceptions and assumptions of PEPs rather than their own perspectives.

One study that has entirely focused on the perspectives of PEPs was that conducted by Watermeyer and Rowe (2021). Their research, conducted with public engagement leads, found that PEPs’ roles were often undervalued and neglected within institutions, subject to academic snobbery and, despite widespread organisational commitments towards engagement, found the emerging profession was often reliant on short-term funding and without a clear career or promotion structure. PEPs’ expertise was often viewed in a very reductionist way, despite them being highly qualified and experienced, meaning teams often felt vulnerable to downsizing or faced assumptions that anyone could step into a PEP role (Watermeyer and Rowe, 2021). Although ethics was not a specific focus in Watermeyer and Rowe’s (2021) work, this may cumulatively suggest not only practical factors that could potentially impinge on ethical good practice (e.g. limited resource/time to allow for ethical consideration) but also prejudicial factors (e.g. where PEPs’ expertise is viewed to be lesser than an academic staff member) in coming to ethical decisions regarding engagement.

While Watermeyer and Rowe’s (2021) work sheds light on the role of PEPs, it also raises potential concerns regarding the ways in which infrastructures, including institutional organisation (Christensen, 2019), funding investment and research assessment, can potentially create a mismatch between what is encouraged and what occurs in practice, at least in the context of public engagement over recent decades. Recently, Davies (2026) has called for more attention from researchers to communicative work ‘in the wild’. That is to say, with an awareness of the complex conditions in which engagement (in the context of science communication) can be influenced not only by its goals, but by the people, organisations, agendas and constraints under which it is undertaken (Davies, 2026).

Here, the recent rise of co-creation, co-production, citizen science or what we might describe more broadly as ‘engaged research’ in the UK has gradually come to influence the work of PEPs. Co-creation, co-production and other participatory approaches are increasingly seeking to involve publics throughout the research process, from the establishment of a project and the development of research questions, through to shaping data collection and the development of outputs, in a manner which often enables expertise and engagement to be distributed throughout the research process (Davies and Avkıran, 2025; Greenhalgh et al., 2016; Mulvale et al., 2024; Pearce et al., 2020). Engaged research is an umbrella term that captures different approaches to embedding engagement into research, but which typically embeds public or community perspectives across the research lifecycle as an integrated aspect of the research, building relationships, sharing power and using inclusive and equitable methods (Wellcome, 2026). These developments potentially alter when and how engagement might take place, but the organisation of such forms of participation like co-creation requires specific abilities and capacities, or ‘expert work’ as Davies and Avkıran (2025) describe and which will likely fall to PEPs. This kind of expert work is not based on technical knowledge and skills alone, according to Davies and Avkıran (2025), but can involve ethical components and is therefore worthy of further investigation.

Given the under-exploration of ethics in engagement contexts, the rising professionalisation of PEPs and their positioning at the intersection of funders’ requirements and the needs of researchers and publics, the data reported here form part of a wider study examining both researchers’ and PEPs’ views on communication and engagement within the fields of science and health research. Here we focus specifically on the PEPs’ data and the research question: how do PEPs communicating and engaging about science and health-related topics consider the ethical dimensions of their communication?

## 3. Methods

INSIGHT was a pilot project, which included three interlinked work packages with data collection taking place in the cities of Bristol and Oxford in 2023–2024. In this article, we utilise one of three work packages, which included 17 interviews with science communication and public engagement specialists at UK academic institutions and in practice settings (museums, science centres, funding organisations and other informal learning spaces). The interview questions covered key themes, such as the role ethics played in their profession, ethical challenges they had experienced and support or resources they utilised. The interview guide is provided as supplemental material. We made a decision not to provide a strict definition of communication and/or engagement for our interviewees, allowing them to operationalise these terms in their own contexts. The research was granted ethical approval from UWE Bristol, and participants were given the option to be acknowledged with the research or to have their input fully anonymised (see supplemental material).

As a pilot project, we concentrated on PEPs mainly based in two geographical areas. Bristol and Oxford both have two universities (University of Bristol/UWE Bristol/University of Oxford/Oxford Brookes). One of the universities, in both cities, is a ‘Russell Group’ university, one of 24 institutions that are research-intensive and provide significant social, economic and cultural impacts across the United Kingdom and around the globe. The second university in both cities is a post-1992 university, a former polytechnic and an institution that tends to have a strong teaching emphasis coupled with research, as well as a much shorter history. We used our connections in these cities and a convenience approach to identify potential interviewees. We compiled a list of people to invite associated with public engagement contexts, including those based in university public engagement teams, local museums, science centres and other informal learning spaces, with the exception of two interviewees who represented funding organisations and were working at a national level but had established relationships and/or funded projects in the two cities. There are limitations in this sampling approach as it may have meant our sample overly represented those who were more likely to be ethically engaged and/or working in particular areas of science and health engagement, but as Bherer et al. (2017a: 3) describe, the ‘invisibility’ of PEPs can sometimes make them challenging to locate, and this allowed us to identify a range of individuals with different roles, settings and levels of experience in relation to engagement in a relatively short time, as was required for a pilot project.

We invited 39 individuals to participate in an online interview, with just under half (43%) agreeing (*n* = 17). The semi-structured interviews were conducted with a range of participants, including two working for funding bodies, nine individuals in university engagement/communication roles, three based in community organisations and a further three interviewees who were museum/science centre based. We recognise that among those we interviewed, different job titles and organisational roles were held, but describe them here as PEPs in recognition that they all worked in or had expertise related to public engagement practice. Interviews were between 20 minutes and 1 hour, with the average interview lasting around 40 minutes, and were conducted between June and September 2023.

Interviews were audio recorded via Microsoft Teams, with notes taken at the time and transcripts checked and annotated in full before being analysed in NVivo 14. We conducted a reflexive thematic analysis focusing on process and meaning, and taking a critical and analytical approach, which included reflection on our own assumptions given that all the authors had relationships, either via academic research and/or practice experience, to the focus of the study (Braun and Clarke, 2022). We conducted six phases in our data analysis process. This involved two researchers familiarising themselves with the data and constructing an initial set of codes and their descriptions, gathering codes into potential themes and reviewing by an external member of our team for repetition and miscoding, and a meeting to finalise themes, codes and definitions before writing up the data.

## 4. Results

In this article, we focus on two themes generated from our data: the *‘*purposes and value of public engagement’ and the ‘evolution of communication and engagement’, in order to address the research question. Both themes included two codes. These are related to ‘the social value/good of communication and engagement’, ‘value of mutuality/dialogue/listening’, ‘drives for engagement/impact/co-production’ and ‘environmental and climate ethics’. Perhaps unusually, rather than taking each theme and code in turn here, we summarise and share elements of this data, with a view to drawing out two key tensions we then identify within the discussion. Although we do not organise the narrative of our results via the coding framework, this is provided in the supplemental information for reference.

### Current context and understanding of engagement

According to the majority of PEPs, engagement is widely considered as a good and valuable thing to do. PEPs either personally aligned themselves with this view or presented this view as a widespread belief they experienced in their professional environment. Sometimes interviewees presented that there was a moral duty to communicate and/or engage, including with those who had contributed to the research.

There is an interesting thing I think about when you’ve had people involved in your research, about letting them know the results of the research, regardless of how involved they’ve been. I think it’s probably just the right thing to do, good ethics with a small ‘e’ um in terms of sharing the findings and making sure they [participants] understand how their contribution [impacted], or they can see what their contribution was part of. (Anon 3, University Public/Community Engagement Representative, Oxford)

They also described situations where public engagement with science can be used instrumentally to achieve particular goals, and/or is considered as good in and of itself. Examples here included when public engagement is used with young people to encourage them to take up a career in STEM, engagement is conceived as ‘a place to bring researchers together with the public’ (Anon 18) or researchers were described as having a ‘moral duty’ to share research with those that contribute to their funding. Such comments were often accompanied by an emphasis on ‘listening’ and understanding potential participants among such processes:

We need to be aware of what communities are there. You know how we are defining communities, whether it’s by geography or by interest or you know, so we really need to be very attuned to what’s around us in order to help researchers. Getting contact with the right type of people. Yeah, and then listening to those communities, what do they need? What do they expect? How they would like to engage with us. (Anon 21, University Public/Community Engagement Representative, Bristol)

### Structural embedding of engagement

It was evident that the widespread adoption of engagement as a key communication means for research was reflected in the institutional structures PEPs had seen set up to promote and embed engagement, either at their own organisations or those they worked with. Such structures existed at research institutions/universities, at funding institutions, as well as in various cultural institutions/social organisations, such as museums. According to some participants, in the recent past, there has been a real push from funders and universities to increase engagement. In some institutions, this fell under moves to promote civic engagement (individual and collective actions to identify and address public concerns) as a central pillar of a university’s activities, alongside teaching and learning and research. In others, it was influenced by a focus on impact in the Research Excellence Framework (REF), the UK’s system for assessing the excellence of research in UK higher education providers, which had generated a rise in ‘impact facilitators’ in a culture of encouraging and increasing engagement. Despite this sense of rising recognition of the importance of engagement, some PEPs still reflected that engagement was not valued in academic institutions on a par with teaching and research, and there was also some questioning as to whether the inclusion of engagement within REF impact generating activities diminishes its visibility and/or encourages a somewhat instrumental approach that utilises engagement for ‘game playing’:

I think there’s some analysis been done of the REF case studies that says people kind of overclaim their responsibility. Which is quite interesting because that’s what they feel the game is. I suppose they feel that’s what the task at hand is. (Anon 15, University Public/Community Engagement Representative, Oxford)

### Evolution of engagement and its ethical implications

The interview data suggest that until recently engagement was often seen as rather unproblematic, that is it was considered to be good in itself, and therefore not resulting in a lot of ethical reflection. There was a sense among PEPs that has now changed, partly because of the volume of engagement activities, which in the participants’ view has increased considerably over recent years, and potentially due to the increasing experience and expertise of PEPs, leading to more critical stances. The rise in both the volume of engagement and awareness of its potential ethical implications means that the ‘mainstream’ ideas of what engagement is and what its purpose should be were being re-defined and even challenged by some participants.

### Engagement as an activity underpinned by values

The majority of PEPs considered engagement, in its ideal form, as an ethical endeavour underpinned by ethical values of respect, mutual benefits, reciprocity and so on. This also includes respect for diverse kinds of knowledge and lived experience, something framed by one interviewee as ‘cognitive justice’ (Anon 21). These are the values that PEPs strive to implement in what they do, and also to impart to researchers with whom they work. PEPs encourage researchers to consider engagement through the lens of reciprocity. This was reflected in the questions that PEPs often described asking the researchers they were working with, such as why engagement should occur in the first place? And what kind of good outcomes would emerge from engagement for publics? This normative sense is that engagement should be beneficial for everyone involved.

Such benefits could take multiple forms. For example, many PEPs discussed the need for engagement to work for the engaged, its participants or publics, and for those being engaged to be at its core – be that school children, students, museum visitors or community members. It should be conducted to a good, professional standard and not take up too much time from the participants. It was flagged that it was tricky to devise engagement so that it is a two-way transaction (and there was some nuance as to whether a transaction meant financial recognition) – but suggestions were that it should be that way.

### Ethics as inherent in engagement

Reflecting these complexities meant PEPs saw engagement as inevitably posing ethical dilemmas at various stages from planning to execution, including what modes of engagement are appropriate and the need to carefully consider audiences and what researchers themselves can or can’t offer. According to one interviewee, engagement is a type of activity inherently underpinned by ethical questions that need to be resolved in practice:

I mean, I feel like it’s [ethics] really integral in theory, but I think the way generally that we approach engagement and try to approach, you know, training and support, supporting others to think about engagement. In theory. It has a lot of aspects to it. That’s asking sort of ethical questions. Probably also even conceptualising engagement and what’s the purpose of it, I feel like there’s a lot of sort of ethical dimensions to that and you know it’s sort of in everything, from thinking about its purpose to who you’re talking to, who you’re not talking to, how you’re engaging with people, whether you’re doing so inappropriate ways, thinking about motives and vested interests and are you including all the people you could be including? Or is it inclusive? Thinking about things from I guess an EDI perspective. Yeah, I feel like it’s [ethics] it’s kind of inherent in the questions we think about when thinking through and planning things. (Anon 19, University Public/Community Engagement Representative, Oxford)

For the PEPs we spoke to, often involved in engagement day in day out, we also saw evidence of ethical consideration as to when and where the sense of a social good or benefit may not be inevitable. One PEP spoke at length about the care they took to both heighten ethical considerations and effectively gatekeep the engagement that took place, particularly when working with people who felt like they needed to be a ‘cheerleader’ for science in the face of being ‘under attack’. This PEP described a sense of needing to ‘temper enthusiasm’ and introduce ‘realism’ which included significant ethical dimensions:

I would say when it comes to thinking about ethics in my field there’s a sort of danger that people think that engagement can only be a social good, and so they just sort of don’t have to worry about it. They’re always doing a good thing. Sometimes my role is to reel people back and sort of ask the question like is this gonna help or is this not going to help? Is this going to do nothing at all or even make things worse? (Anon 2, University Public/Community Engagement Representative, Oxford)

This sense of needing to focus on the lasting impacts for participants, including if engagement would empower communities, or if communities had agency to change a situation based on research (e.g. around environmental behaviours) was noted in a number of comments by PEPs:

So, when we talk about benefit, engagement teams have started considering new kinds of questions: Is there benefit for whom and how? How that benefit will be shared equitably? (Anon 21, University Public/Community Engagement Representative, Bristol)

Do you want people to just leave even more in a state of despair? . . . What’s the purpose of it [engagement], really? By presenting people with data and information, how are you also supporting them to take action on things? (Anon 17, Community Organisation Representative, Bristol)

### Engaged research

Rising interest in ethical issues underpinning engagement also appeared to be prompted by the current focus on one particular form of engagement, that associated with co-creation, co-production and/or engaged research. This kind of engagement is being promoted by some funders in the UK and, as the PEPs highlighted, this shift brings a new set of ethical considerations for both researchers and practitioners.

In some cases, this shift to engaged research represented a sharpening of the desire for a reciprocal relationship between researchers and publics but extended this further to an ethical consideration of both what was being consented to and how contributions were recognised:

When engagement practitioners try to involve publics on a specific issues, they have an ethical responsibility to be clear about what they are going to do with publics . . . In practise, a lot of the time, you see these opportunities and people want to engage with them but there’s not a lot of clarity about what’s going to happen with those ideas when they’ve been presented. (Anon 23, Museum/Science Centre Representative, Bristol)

However, this re-shaping of engagement could also fundamentally alter when ethical considerations might arise in the timeline of research. One PEP described how, until recently, ethics had not featured front and centre in engagement activities, as ‘historically’ engagement tended to be the thing that happened at the end when results were ready to be shared, and this meant for this interviewee, they did not think ethics had been particularly well considered in the past. They went on to describe what they saw as a shift away from the idea of ‘mass’ engagement (as opposed to engagement which might target specific communities or embed engagement), which they perceived had become less desirable among funders, and had been pushed out from funding schemes, relying more on funding from universities and research institutions. Instead, they said funders often wanted to see ‘engaged research’.

### Emerging ethical dilemmas posed by engaged research

Encompassing engagement within research clearly offered significant opportunities according to our PEPs, when done effectively, but questions were also raised about where this left the support for wider engagement initiatives, how much researchers actually intended to undertake engagement themselves through these kinds of models (vs whether they simply sub-contract it out to PEPs and/or others) and how decisions were weighted when deciding on where funding should be awarded (is the decision based on the research, engagement or both?). There was an ethical question as to whether engaged research is appropriate as a default form of engagement. One PEP saw it as a minority practice, partly as people were still coming to understand how to do it effectively, but also because for much research it may not be relevant, as they described ‘I don’t think we should be forcing square pegs into round holes here, we just need to do the appropriate form of engagement with all the appropriate thinking for the activity in hand’ (Anon 3).

A very tangible ethical issue raised was that it was now not always clear if participatory forms of engagement, with a ‘blurring of boundaries’ between public engagement and engaged research, required a formal ethical process or ‘safety net’. This diffusion of engagement throughout all research described by one PEP as a ‘greater kind of integration of society and research’ (Anon 21) was leading to questions for organisations as to how activities could be supported and sustained. As this PEP continued to describe, there was no plan to grow the engagement team at their university, so they were thinking about how to conduct activities in an environment of greater need, while ensuring quality:

We need to be thinking how to streamline our advice, which is why we started already sort of doing these asynchronous resources like little videos and guidance and resources that researchers can access in their own time and then come and talk to us certainly when it’s really beneficial. (Anon 21, University Public/Community Engagement Representative, Bristol)

### Avoiding extractive engagement

A further point highlighted by PEPs was that engagement undertaken within engaged research should absolutely avoid being ‘extractive’, an area where there were clearly some important dimensions to consider. These included how to ensure communities were sustainably supported. One interviewee described there being a risk of ‘instrumentalising participants’ (Anon 3) in engaged research, while a further PEP noted ‘you can’t do that kind of work short term’ (Anon 18). Without proper funding, short-term engagement with communities in engaged research was perceived to have significant consequences:

If you’re [as a researcher] asking to work with a community, giving them money and then you disappear, they’ve gone back to where they were. They got attention for a bit and not anymore and I think I think the ethics of that are really problematic. I think that if you want to do community work, with communities who are struggling, you’ve got to do it in an extended way . . . It has the potential to be more negative than positive. (Anon 18, Museum/Science Centre Representative, Oxford)

As already touched upon, some PEPs we spoke to were concerned about how to best support a more diffused model of research embedded engagement, while others were also worried about the potential impact on PEPs themselves. The PEPs who had built their careers around approaches that were now shifting away from funders prioritisations expressed concern about the impact this could have in terms of loss of jobs and expertise when:

. . . these big funders just veer suddenly . . . You’re losing expertise even those senior folk who’ve been around a long time and then you’re just left once again trying to build that up in five years’ time. (Anon 2, University Public/Community Engagement Representative, Oxford)

### Equity, diversity and inclusion

Concepts and/or values of equity, diversity and inclusion (EDI) appeared to be shaping engagement activities to an extent perhaps not seen before. This was reflected in increased awareness of the need to consider EDI in the design and execution of engagement. Here, PEPs gave examples, including consideration of environmental impacts in communication choices, through to the ethical representation of indigenous voices when curating exhibitions, and consideration of any ‘cultural sensitivities’ that need to be considered in advance of engagement.

There were two key manifestations of this underpinning relationship between EDI and engagement. The first being around the consideration of fairness, whereby engagement was tied to the notion of (social) responsibility and accountability. A multitude of examples were offered here that ethical engagement should not be exploitative, that people should be fairly rewarded for their time and input, and once again a reiteration that ‘public engagement needs to be a conversation’ (Anon 1, University Public/Community Engagement Representative, Bristol). Here, more than one PEP described the need for such positive outcomes to also be reaped by researchers, as if engagement is to be beneficial to all involved, researchers should also benefit from engagement, in terms of new knowledge gained from interacting with publics, new perspectives or understandings. In the words of one participant, the practice of reflexivity, which should be inherent in engagement activity, can help researchers and engagement professionals to become better citizens.

Obviously the best outcomes are when the researcher, for me when the researcher, one of the bits of feedback, is when the researcher takes away that it’s made them think differently about their research by talking to the public and not just that they ticked a box and spoke to X number of people. (Anon 18, Museum/Science Centre Representative, Oxford)

PEPs clearly identified researchers as participants themselves, rather than this being a notion reserved for publics, but we also noted some questioning of the value of engagement for researchers. This included concern as to whether some researchers involved in engagement really benefitted from it when other aspects of their job roles might offer better recognition, or that their careers could be harmed as time, attention and resources are diverted from research.

We’ve talked about diversity and I think it’s just a big problem because the engineers that we’re involving, we’re wanting to be from underrepresented groups and I think it’s a big kind of ethical dilemma. I don’t know, women for example, women engineers have kind of disproportionately taken the load on public engagement work . . . We obviously want underrepresented groups, and we want engineers to be involved with outreach and public engagement but it’s just making sure that they’re paid correctly and they’re not overworking themselves, that kind of thing. (Anon 26, Funder Representative, National)

I think it [engagement] can often take more time than it’s ethical to take from researchers, actually, and I think they don’t necessarily know what they’re signing up for and that can really increase stress on somebody who’s already very stressed in difficult work environment. (Anon 18, Museum/Science Centre Representative, Oxford)

### Social justice

The second manifestation of this thinking related to EDI and engagement was around the way engagement can be an activity geared towards social justice, an ethical goal in itself. For some participants, engagement is not only inherently ethical (in an ideal situation) but also geared towards addressing current and past injustices. For example, engagement was being used in a museum to expose and address the injustice and silences of colonial science. An arts-organisation representative described ethics as embedded in their organisation’s engagement practice and delivered as social justice. Another example involved a charity aiming to increase engagement of publics with the natural world – activities that were attuned especially to engaging marginalised communities traditionally excluded from such engagements, such as people from ethnic minorities, different socio-economic backgrounds and with disabilities. Such experiences could involve ethical dilemmas, including considerations as to how museums/galleries could be harmful/unwelcoming spaces for excluded communities and ways to engage with publics in an ethical and justice-orientated way. However, this emphasis on social justice marks an interesting shift – in some previous scholarship, the goals of engagement/communication tended to be more closely aligned with the goals of scientists promoting their research, whereas this marks a move to address and consider broader societal issues.

## 5. Discussion

### Tension in the data: Redefining engagement through ethics (i.e. contesting the notion that engagement is always a virtue/necessary/ethical)

An interesting tension arises in the data at the intersection of two trends. On the one hand, the promotion of engagement understood as a force for good has led to a wider adoption of engagement and its greater popularity. On the other hand, the rising number of engagement activities has resulted in an increased awareness of the need to ethically scrutinise them. This in turn had prompted some of our interviewees to offer a critique of assumptions surrounding the practice of engagement. What emerges is a more nuanced account of what engagement is and how it is done in practice, highlighting the potentially untapped input of these professionals and their expertise to academic research (Besley et al., 2021; Chilvers, 2017).

While committed to the idea of engagement, some PEPs sought to dispel the pre-conception that engagement is always ethically neutral, unproblematic and/or desirable. This is linked to the fact that while science in itself can be a force for good, it can also be harmful. Engaging publics with such science would not always be beneficial to them or serve public interest (Brunner, 2017). PEPs questioned the need to avoid being ‘cheerleaders’ for research and a questioning of the goals of some engagement initiatives, for example those that unquestionably presented the value of a scientific career.

Here we saw a glimpse of the role of funders and their funding priorities, whereby engagement is extensively influenced by what the funders want to be implemented. As funders help shape the goals that engagement should achieve, opportunities to probe the ethical dimensions associated with such goals can potentially become secondary, perhaps best summed up by this quote:

You know, it’s all very well for us to talk about the ethical problems, but if the funders expect certain kinds of engagement and reward certain kinds of engagement, then that’s sort of what we have to do. (Anon 2, University Public/Community Engagement Representative, Oxford)

Although researchers often argue that publics have the right to know what scientists are doing, information about scientific activities and research does not always need to be shared according to some PEPs if this does not result in tangible benefits for the publics. There should be more reflection on why research should be shared and publics engaged. Benefits stemming from engagement for researchers should not be conflated with the benefits for publics. Both points reflect long-standing concerns regarding the potential of expanding technocracy (Chilvers, 2007, 2017) or tyrannies of participation (Cooke and Kothari, 2001).

It was also apparent that engagement can help researchers’ careers but can also harm careers, as noted in prior research (Watermeyer, 2015). An interesting observation stemming from the data is that PEPs identify scientists as participants of engagement and just as questions were raised about impacts on participants, so too were there concerns about the impacts on researchers. At times, this is related to the ongoing lack of recognition of engagement within institutions, including via promotion mechanisms. Though REF could potentially heighten support for engagement, it raised critical ethical reflections as to how engagement is presented in such contexts, including how research and its engagement produce positive change (Watermeyer and Rowe, 2021) or could contribute to a kind of ‘marketisation’ of engagement (Barry and Legacy, 2022).

These kinds of critical reflections on career and research outcomes also extended to ethical dimensions about the role of PEPs themselves. The rise of an engagement agenda was essential for facilitating funding, yet these same infrastructures could be a potential obstacle for progressive engagement and held the power to radically change PEPs’ working contexts at any time. Some interviewees questioned how funders keep up with contemporary approaches, when there is a tendency to rely on experienced academics advice, some of whom could be out of touch with engagement, yet influenced ‘the big strategic decisions’. While others highlighted that if staff with significant engagement experience simply left a funder, this could result in engagement almost entirely disappearing from prioritisation.

Thus, there was a sense that there may not be as much experience with nuanced engagement in some funding contexts and what it can potentially provide, and that potentially the expertise of experienced PEPs to contribute here is overlooked (Watermeyer and Rowe, 2021). This led to further ethical questions as to how PEPs are sustainably resourced (when they often rely on short-term funding and/or contracts) and the lack of clear routes for their own career progression, highlighting ‘structural forces’ impinging on PEPs’ experiences (Pellegrini et al., 2025: 20), organisational aspects which may make PEPs’ working contexts more fragile (Bherer et al., 2017b). The role of funders in influencing scientific research agendas has long been discussed (Besley et al., 2021) and has its sensitivities, but here we see some specific apprehension associated with engagement explicitly (Davies, 2026) and which suggests an ethical dimension. Similarly, while previous research has pointed to the negative career implications for researchers undertaking engagement (Watermeyer, 2015), here we find similar reservations for PEPs despite their ultimate support of engagement’s importance and their valuing of funders’ investments.

Overall, the PEPs’ critique of engagement seeks to redefine engagement as more ethical, equitable and attuned to the publics’ needs or perspectives rather than just following the agenda of researchers, or their funding organisations. It is a striking departure from the deficit models that implied a one-way communicative relationship and some definitions of engagement that tend to be centred predominantly on scientists’ goals, but nonetheless raises ongoing ethical questions.

### Tension in the data: Reshaping the landscape of engagement – the rise of engaged/co-produced research and its ethical implications

As discussed above, the first shift in engagement activities and practice noted by the PEPs is the increased awareness of the ethical implications of engaging publics. Another shift is the increased focus by funders on a particular kind of engagement, described as engaged research, co-creation or co-production of engagement and so on. This shift is reflected in the increased funding for engaged research and in what our interviewees perceive as a decrease in the funding of other types of engagement, especially in the area of medical and health-related research.

These new types of engagement are being championed as more desirable, with a clear underpinning ethical imperative in bringing research and engagement closer in proximity, closing the loop between engagement and its impact, and with a perception that there is now more funding for engaged research compared to some of the more generic engagement initiatives that were funded in the past. One interviewee highlighted this kind of redefinition of engagement as somewhat inevitable, an ‘evolving’ of a relatively new field continuing to establish its usefulness on the peripheries of more widely valued and respected research. What was less clear is how PEPs and their institutions can support this rise in engagement and ensure it is carried out ethically, appropriately and with underpinning resources, including understanding whether ethical review is required. There was a sense of needing to do more with less among some PEPs, as well as a recognition (among both funder representatives and PEPs) that they were still understanding and learning about the implications of such changes.

The rise in prominence of co-created, produced or engaged research thus brings with it a host of new ethical issues and considerations that were highlighted by PEPs. While interviewees were broadly supportive of this mode of engagement, they cautioned around various aspects of the promotion of this type of engagement from an ethical perspective, including the potential loss of jobs and expertise with a more diffused model or re-branding of engagement, and whether engaged research should be seen as the default option. Perhaps most significant, however, was some of the potentially negative impacts on participants if this were to inadvertently encourage a more extractive form of engagement. In this sense, as one participant summed up, ‘the ethics piece is going to be bigger than the more traditional [engagement] stuff’ (Anon 3). We noted many instances where PEPs were driving ethical good practice, motivated by a general sense of morality and civic duty (Brunner, 2017) and/or care (Davies and Avkıran, 2025) as opposed to upholding a pre-determined professional code of ethics.

This tension between being supportive of the goals of engagement, while wary of the potential impact, was reminiscent of a point highlighted in work by Barry and Legacy (2022), who describe those working in public engagement facing two conflicting narratives. On the one hand, there is the ‘virtue’ of public engagement as an ethical vocation promoting values around justice, democracy and representation. On the other hand, there is the narrative of professionalisation, which requires PEPs to market their skill sets and compete for work in an emerging context of professional standards. Where our data perhaps differs from Barry and Legacy (2022) is in the ways in which the PEPs we spoke to were both driving their own professional standards and occasionally expressing muted dissatisfaction as to the lack of status and respect that may usually go hand in hand with enhanced professionalisation (Brunner, 2017). Here we see a kind of ‘mood of tolerance’, as described in Watermeyer and Rowe’s (2021: 1304) observations of PEPs, whereby PEPs’ commitment to the virtues of their work meant they appeared to tolerate some of these problematic aspects in terms of their professional standing. Lee (2014) noted that public engagement practitioners in seeking to ‘walk the talk’ regularly engage in reflective practice, deliberation and the reduction of power imbalances to the extent that they may in some ways disempower their own expertise, though our interviewees appeared very happy to express their views in the context of our interviews.

It was interesting to see that the engaged research approach (encompassing techniques like co-creation and production), often discussed in the literature in relation to democratic values, has come to be perceived as the preferred format of engagement among funders, at least in the UK. However, our data point to some significant differences in the ways in which funders and professionals understand the value and purpose of this type of engagement, as well as the need for ongoing ethical consideration.

## 6. Conclusion

Returning to our research question, how do PEPs communicating and engaging about science and health-related topics consider the ethical dimensions of their communication? We find complexity, tensions and significant ethical insight within our data. This suggests that PEPs can indeed usefully contribute to and advance academic discussion around engagement, including more recent approaches towards ‘engaged research’, moving beyond the limitations of a focus on aspects like scientists training and researchers’ views towards engagement alone (Besley, 2020; Besley et al., 2021). The data also highlights PEPs’ expertise not only as intermediaries but also as communicators and listeners, possessing skills in reflective practice, deliberation and a deep awareness of cultural and social sensitivities, among other capabilities. Moreover, this expertise often positions them to perform a role akin to that of an ethical advocate.

Our study has limitations. We found a good degree of receptiveness to engagement in interviews, but our focus on two cities and use of existing networks means our findings may not be taken as granted in other national or international contexts. Previous research in this space has found the openness to discussion of engagement to be highly institutionally dependent, with aspects like institutional contexts and local politics all playing a role (Watermeyer and Rowe, 2021). It is likely that PEPs who either do not perceive ethics to be of value to their role or feel they lack expertise on this topic would not have responded to a request to interview. Therefore, it is possible that our interviewees represent those with greater confidence in their expertise. Our use of the term PEP may also distort the likely variations in experience among these professionals (Bherer et al., 2017a; Escobar, 2011). Finally, our interview work was conducted in 2023–24. We have subsequently witnessed a number of funders and organisations investing enhanced time and consideration in the emerging ethical issues associated with engaged research, including the development of some supporting mechanisms and resources. For example, the Institute for Community Studies and UKRI (2024) have been driving support of ethics in citizen science settings, the University of Oxford (2025) has recently introduced a Responsible Knowledge Exchange, Engagement and Impact (RKEEI) Framework, and others are highlighting the need for ethical consideration when working to develop societal impacts from research (e.g. Jensen et al., 2025). Supporting mechanisms for ethically engaged research were also an area we discussed with our interviewees, and which is forming the basis for a future article.

Our research findings mirror previous work that has suggested community engagement practitioners face contradictions in their work and ‘serve’ two masters ‘the organisations that employ or contract them and the communities whose views they are engaged to elicit’ (Christensen, 2019: 196; Lee, 2014), as well as a range of other paradoxes. It also suggests there is a need for further collaboration, and potentially less siloed approaches between those influencing engagement infrastructures, researchers and PEPs (Holmes et al., 2019), recognising engagement involves multiple participants (Barry and Legacy, 2022) in an ongoing process of negotiating different forms of expertise (Davies and Avkıran, 2025). Finally, it repositions the conversation as to gaps between research and practice, strongly suggesting that further research *with* practitioners is beneficial for academic understanding of communication, engagement and engaged research.

## Supplemental Material

sj-docx-1-pus-10.1177_09636625261440854 – Supplemental material for Public engagement professionals: Exploring ethical tensions in communication, engagement and co-creation Supplemental material, sj-docx-1-pus-10.1177_09636625261440854 for Public engagement professionals: Exploring ethical tensions in communication, engagement and co-creation by Clare Wilkinson, Aleksandra Stelmach, Michael Parker and Milly Farrell in Public Understanding of Science
