# Supplementary material for: Public engagement professionals: Exploring ethical tensions in communication, engagement and co-creation
Source: Public Underst Sci. 2026 May 5;35(6):728–46. doi: 10.1177/09636625261440854 (PMC13380656; doi:10.1177/09636625261440854)
Supplement: sj-docx-1-pus-10.1177_09636625261440854 – Supplemental material for Public engagement professionals: Exploring ethical tensions in communication, engagement and co-creation [file sj-docx-1-pus-10.1177_09636625261440854.docx]

**Public Engagement Professionals: Exploring Ethical Tensions in Communication, Engagement and Co-Creation**

**Prof. Clare Wilkinson (corresponding author)**

Science Communication Unit, UWE Bristol, Coldharbour Lane, Bristol BS16 1QY, United Kingdom

+44 (0)117 328 2146

[Clare.Wilkinson@uwe.ac.uk](mailto:Clare.Wilkinson@uwe.ac.uk)

**Co-Authors**

**Dr Aleksandra Stelmach**

Department of Management, The University of Exeter Business School, University of Exeter, Rennes Dr, Exeter EX4 4PU

[A.Stelmach@exeter.ac.uk](mailto:A.Stelmach@exeter.ac.uk)

**Prof. Michael Parker**

Ethox Centre, Nuffield Department of Population Health, Big Data Institute, University of Oxford, Oxford OX3 7LF, United Kingdom

[michael.parker@ethox.ox.ac.uk](mailto:michael.parker@ethox.ox.ac.uk)

**Milly Farrell**

Ethox Centre, Nuffield Department of Population Health, Big Data Institute, University of Oxford, Oxford OX3 7LF, United Kingdom

[milly.farrell@bdi.ox.ac.uk](mailto:milly.farrell@bdi.ox.ac.uk)

**Contents:**

Participant Information Sheet p. 1-5

Consent Form p.6

Interview Questions p.7

Coding Framework p.8-12

**Participant Information Sheet**

**INSIGHT: Ethical Best Practice in Science Communication and Engagement**

***What is the project about?***

[INSIGHT](https://www.uwe.ac.uk/research/centres-and-groups/scu/projects/insight) aims to identify how researchers and practitioners communicating and engaging about science and health related topics consider the ethical dimensions of their communication. By conducting two advisory workshops and two focus groups (one in Bristol, one in Oxford) we will understand more about the role that ethics plays in communication and engagement with research. A series of interviews in UK academic institutions, and in museums, science centres, and other informal learning spaces, will contribute to the project gathering UK-based evidence on the ethical dimensions of science communication and public engagement with science and health topics. The project is being led by Dr Clare Wilkinson, Co-Director of the [Science Communication Unit](https://www.uwe.ac.uk/research/centres-and-groups/scu/), UWE Bristol in collaboration with Professor Mike Parker, Director of the [Wellcome Centre for Ethics and Humanities (WEH)](https://www.weh.ox.ac.uk/)/[Ethox Centre](https://www.ethox.ox.ac.uk/), University of Oxford.

INSIGHT is exploring three questions over the duration of the project:

1. What are the key ethical considerations that need to be made in science communication and public engagement contexts, what frameworks exist and where are the gaps?
2. How do those communicating and engaging around science and health (researchers and practitioners) consider the role of ethics in their activities?
3. What support is in place for researchers and practitioners to assess and ascertain the ethical dimensions of their communication and engagement practices?

INSIGHT: Ethical Best Practice in Science Communication And Engagement (SRG22\220481) has been funded by a BA\Leverhulme Small Research Grant, supported by the Leverhulme Trust.

***What the research will involve?***

It is your choice to agree to participate in this research and you may have been invited to participate in one, or more, of the following data collection methods. This project is seeking to be inclusive and ensure diversity of representation. The email or advert you have responded to will indicate which **method is relevant to you**:

1. **Advisory Workshops** will last 1.5 to 2 hours, during which we will facilitate a group conversation with invited participants who have experience or expertise related to the focus of the project. The first workshop will help us to understand the current ethical landscape of science communication, where ethical considerations are made and where gaps lie, and to understand how we can best share the findings of INSIGHT to have impacts. The second workshop will be an opportunity for us to share key findings with participants, working with them to understand the implications of our findings and ascertain a set of key recommendations for the sector from the project. It is your choice whether to participate in both workshops and there will be an opportunity to withdraw from data collection between workshop 1 and workshop 2. The workshop will be audio recorded and we will be conducting them online via Teams.
2. **Focus Groups** with researchers, who are communicating and engaging around their research, will explore how researchers consider the role of ethics in their activities, any support and guidance that is in place and if they consider the role of communication/engagement in broader ethical approval processes. We will share a set of ground rules at the outset of the Focus Group and participants will be requested not to share information from the Focus Groups after they have taken place. You may leave the Focus Group, or decide not to answer a question, at any time. The Focus Group will be audio recorded and we will be conducting them either online via Teams, or in person at a university, or meeting room, in Bristol or Oxford. Focus Groups will last 1 to 1.5 hours and include a short comfort break with refreshments.
3. **Semi-structured Interviews** that will last for 30 to 45 minutes with science communication and public engagement specialists at UK academic institutions and in practice settings (museums, science centres, and other

informal learning spaces) will allow us to identify the support they offer researchers and practitioners and any frameworks that are utilised for the consideration of ethical issues. We have designed our interview questions sensitively, and you may stop the interview, or decide not to answer a question, at any time. The interview will be audio recorded and we will be conducting them either online via Teams, in person at the university, in a semi-public place or at your place of work.

If you are under 18, please let us know as we are only able to talk to over 18’s in this project.

***What are the possible benefits of taking part?***

The information we get from you will be important as at present researchers and practitioners often undertake science communication and public engagement activities with limited ethical guidance, which may also result in limited assistance when ethical issues then arise. At present, to our knowledge, there has been limited primary data collection on this topic, despite ethical, legal and social issues (ELSI's) associated to science itself often being a focus of activities in this area, and also rising numbers of programmes training both science communicators and public engagement specialists. We hope INSIGHT will create a space to consider some of the ethical ramifications of communication, including questions it raises about power, access, purpose, autonomy, privacy and values.

***What are the possible disadvantages and risks of taking part?***

We do not anticipate any significant risks or disadvantages from taking part. You will have the option of stopping at any point of the Advisory Workshop, Focus Group or Semi-Structured Interview and should you experience any negative emotions after taking part, you can request to withdraw your data from the Focus Group or Semi-Structured Interviews. Unfortunately, this won’t be possible for the Advisory Workshop.

We will be asking questions that may relate broadly to your working contexts, organisations, research and/or practice, therefore you may be concerned about risks to your position of employment, or wider networks. You will not be required to give specific examples, and if you prefer to participate anonymously, there is the option to do so, and your anonymity will be protected.

Very rarely we may be required to share confidential personal information without consent if we are required to do so by statutory law. This may happen if we have safeguarding concerns about a vulnerable adult or young person. The research will follow the guidance of the [UWE Bristol Safeguarding Policy](https://www.uwe.ac.uk/life/health-and-wellbeing/staying-safe-on-and-off-campus/safeguarding). If you have personal concerns related to the public interest, then this [website](https://www.gov.uk/whistleblowing) also offers support and advice.

***What if I don’t want to take part?***

You are under no obligation to take part in this study.

***If I take part, what information will you collect about me and how will this be stored?***

We will collect your name, contact details, and some information on how long you have worked in your role and where you work. All data will be held according to General Data Protection Regulations (GDPR). Further details are given in the Privacy Notice which accompanies this information sheet.

***Will I be anonymous and can I remove my data?***

As participants in this research are participating in a professional capacity you will have the option to have your contributions anonymised or to be identified with your comments. If you decide to remain anonymous, your comments will not be attributed to you and we won’t reveal your identity when we write up this research unless you choose to be identified. The recordings from the Advisory Workshop, Focus Groups and Semi-Structured Interviews will be used for research purposes only and they will be destroyed after we have written them up. Those written files will then be stored on a password protected file that is available only to the researchers. All personal data associated to this project will be destroyed by 31^st^ July 2030. If, following data collection, you decide you no longer want to participate in the research you can contact us at the address below by the **15^th^ February 2024** and we will remove your comments. We are only able to offer this option for the Focus Group and Semi-Structured Interviews, and it does not apply for the Advisory Workshop.

***What will happen to the results of the study?***

The results of the study will be used to help us design a larger funding application. We may also share the results at academic conferences, in an academic journal article and a book. We will be creating an open access digital resource as a result of the advisory workshops and plan an online webinar at the later stages of the project. We will send you a copy of the summary of results if you wish.

***How do I take part?***

If you would like to take part in the project, the researcher who has contacted you will provide details of when the Advisory Workshop or Focus Groups are taking place. If you have been invited to participate in a Semi-Structured interview, they will contact you to arrange a convenient date or time. You can either provide a telephone number/email address for us to contact you via the phone, or we can send you a link to connect online. To speak to us online you will need to have an audio function on your phone, computer or tablet and it is your choice whether to turn on your webcam.

***What if I agree to take part and there is a problem or I change my mind?***

If you have opted to take part, but later change your mind for any reason, you can withdraw from the project before the data collection takes place by contacting us directly, or at any point during the Advisory Workshop/Focus Group/Semi Structured Interview by telling the researcher. If you wish to withdraw your Focus Group or Semi-Structured Interview data after you have participated, please contact us by **15^th^ February 2024.** If you have a concern or wish to complain about any aspect of the project, please contact us at the address given below.

***How to contact us and further information***

For further information, questions about the project, or to withdraw your data, our contact details are as follows. Please refer to the ‘INSIGHT’ project when you contact us:

Dr Clare Wilkinson

Science Communication Unit

Telephone: +44 (0)117 32 82146

E-mail: [clare.wilkinson@uwe.ac.uk](mailto:clare.wilkinson@uwe.ac.uk)

This project has been approved by the UWE Bristol Ethics Committee [ref no: HAS.23.02.083]. You can contact them at: [Researchethics@uwe.ac.uk](mailto:Researchethics@uwe.ac.uk)

**Consent Form**

**INSIGHT: Ethical Best Practice in Science Communication and Engagement**

This consent form will have been given to you with the Participant Information Sheet. Please ensure that you have read and understood the information contained in the Participant Information Sheet and asked any questions before you sign this form. If you have any questions please contact a member of the research team, whose details are set out on the Participant Information Sheet

If you are happy to take part in an Advisory Workshop, Focus Group or Semi-Structured Interview please sign and date the form. You will be given a copy to keep for your records.

- I have read and understood the information in the Participant Information Sheet, dated 28/02/2023, Version 0.1, which I have been given to read before asked to sign this form;
- I have been given the opportunity to ask questions about the project;
- I have had my questions answered satisfactorily by the research team;
- I agree to the information I provide being used for research purposes, including publications, and agree that quotes may be used in research outputs including the final report of the study. These quotes will be anonymised if that is my choice;
- I understand that my participation is voluntary and that I am free to withdraw at any time until 15/02/2024 if I have participated in a Focus Group or Semi-Structured Interview, without giving a reason;

I agree to take part in the research

I agree to be identified with my quotes and contributions

Name (Printed)………………………………………………………………………….

Signature……………………………………………………. Date…………………….

**Interview Questions**

- Could you please tell me a bit about your role at **XXX** [Institution’s name] and how it is related to communication and engagement?
- In your experience, what role does ethics play in your professional field?
- Please explain why it is so.
- In your professional capacity, have you or your colleagues been faced with issues related to communication and engagement work that required considerations of ethics?
- If yes, could you please describe the issue and how it was addressed?
- Are there any particular guidelines or advice on ethics that have been adopted by your employer/institution or that are required in your field?
- If yes, could you please describe what they relate to?
- If no, would it be useful to have such advice and in which areas?
- In your view, what kind of ethics support should or could be provided by employers/institutions in your field?
- What should be the role of ethics committees in relation to communications and engagement activities?
- Do ethical issues ever impact on the collaborators you might be working with on communication or engagement activities?
- In your view, are there any needs in terms of training or resources about ethics that should be addressed in your field?
- Are there any challenges related to ethics that could affect your field in the future?
- Would you like to add anything to this conversation? Perhaps we have overlooked a topic or you would like to share a reflection on an issue related to ethics with us?

| Public Engagement Professionals: Exploring Ethical Tensions in Communication, Engagement and Co-Creation  Coding Framework [15/04/2024] | | | |
| --- | --- | --- | --- |
| Theme | | Code/s | Description/Summary |
| 1. Purposes and Values of Communication and Engagement | | 1.1 Social value/good of communication and engagement | *Communication and engagement assumed as inherently ethical in their values* |
|  |  | 1.2 Value of mutuality/dialogue/listening | *Engagement approaches create spaces for mutual learning, engagement and listening* |
| 2. Evolution of Communication and Engagement | | 2.1 Drives for communication, engagement, impact and co-production | *Increase in participatory approaches, including at early stages of the research process, but questions over where the capacity is to support this. Impact generation (including capturing in REF) as a driver for communication and engagement activities. Co-production increasingly part, rather than separate to the research process.* |
|  |  | 2.2. Environmental and climate ethics | *Increasing need to consider environmental impacts in communication and engagement activities* |
| 3. Ethical Issues in Communication and Engagement | 3.1 Relationships | 3.1.1 Trust/Hierarchy/power | *How the building and breaking of trust creates ethical dimensions in communication and engagement.*  *Different participants in the process holding different relationships and access to power* |
|  |  | 3.1.2 Longevity/legacy | *Communication and engagement often happen in short cycles, but relationships and their impacts take time* |
|  |  | 3.1.3 Generosity | *The gift of volunteering, undertaking emotional labour as being essential to communication and engagement processes* |
|  | 3.2 Content | 3.2.1 Science and Health as ethical issues | *Science and health can be controversial, polarising and contain ethical topics inherent to research* |
|  |  | 3.2.2 Exclusion | *Certain science and health topics would be unethical to communicate and/or the organisations that support them (e.g. military research, tobacco companies)* |
|  |  | 3.2.3 Framing | *Ethical dimensions of distorting, creating narratives, diverting from fact, lacking transparency* |
|  |  | 3.2.4 Processes and practicalities | *Different methods (e.g. online/offline) and processes (e.g. paying) having different ethical burdens* |
|  | 3.3 Implications | 3.3.1 Positive impacts of communication and engagement | *The ethical benefits of participation in communication and engagement, including for all participants* |
|  |  | 3.3.2 Harms for participants | *Burdens and harms for public participants, such as psychological, emotional and financial* |
|  |  | 3.3.3 Harms for researchers | *Burdens and harms for researchers, such as negative media coverage, online trolling and negative career impacts* |
|  |  | 3.3.4 Harms for practitioners | *Burdens and harms for practitioners e.g. negative reactions to front of house staff, emotional labour in anticipating ethical aspects* |
|  |  | 3.3.5 Loss of control | *How communication and engagement can be misrepresented or cause reputational damage* |
|  | 3.4 Culture Change  (also connects to codes under Theme 2) | 3.4.1 Purposes matching experiences | *Communication and engagement needing to match its intentions with actions (e.g. not PR in disguise)* |
|  |  | 3.4.2 Changing political landscapes | *Changes around acceptability of language/terminology and ‘cancel culture’ and the ethical considerations raised* |
|  |  | 3.4.3 Decolonisation | *Awareness of the role of communication and engagement in decolonisation, including the roles played of institutions in the cities of Bristol and Oxford* |
|  |  | 3.4.4 Equality, Diversity and Inclusion | *Ethical dimensions of who is missing in communication and engagement approaches, why and how* |
| 4. Ethical underpinning of the functions of communication and engagement | | 4.1 Relationship to research ethics | *Assumption that communication and engagement is covered by research ethics processes and/or lack of awareness as to if it is covered* |
|  |  | 4.2 Strategic and practical institutional support for communication/engagement | *Institutional and practical commitment to consider communication/engagement, including its ethical dimensions* |
|  |  | 4.3 Lack of ethical underpinning | *Lack of visibility of communication/engagement in ethics processes, inappropriate timings, practicalities meaning its not fit for use* |
|  |  | 4.4 Role of evaluation | *Assumption that ethical aspects are covered if evaluation methods are in place and/or that there is a relationship between ethics and evaluation* |
| 5. Responsibility for Ethics | | 5.1 Universities | *University responsibilities, advice or accountabilities with regards to communication and engagement* |
|  |  | 5.2 Funders | *Funding bodies responsibilities, advice or accountabilities with regards to communication and engagement* |
|  |  | 5.3 Researchers | *Researchers’ responsibilities, advice or accountabilities with regards to communication and engagement* |
|  |  | 5.4 Practitioners | *Practitioners’ responsibilities, advice or accountabilities with regards to communication and engagement* |
|  |  | 5.5. Publishers | *Publishers’ role in provoking ethical considerations in communication and engagement* |
|  |  | 5.6 Communities/external organisations | *Community group and other organisations roles in provoking ethical considerations in communication and engagement* |
|  |  | 5.7 Co-creation | *Ethical responsibilities require co-created models* |
|  |  | 5.8 Ethics as a grey area | *Not knowing who holds responsibility/accountability* |
| 6. What could ethics in communication and engagement look like? | | 6.1 Person focused | *The need for advice and support from people rather than documents/guidance* |
|  |  | 6.2 Training | *What would constitute ethics training and how does this relate to existing training gaps?* |
|  |  | 6.3 Outward looking | *Guidance ‘under development’, needing to be collaborative, or drawing on other resources/disciplines (e.g. restorative justice)* |
|  |  | 6.4 Resources | *Case studies, digital resources, bite-sized content, codes of practice* |
|  |  | 6.5 Commensurate | *Need to be appropriate to time, recognition and budget for communication and engagement* |
|  |  | 6.6 INSIGHT | *Comments related to involvement in focus groups and interviews being beneficial and/or a prompt to think about ethics* |
